# Cost-Effective, Highly Selective and Environmentally Friendly Superhydrophobic Absorbent from Cigarette Filters for Oil Spillage Clean up

**DOI:** 10.3390/polym10101101

**Published:** 2018-10-05

**Authors:** Qiancheng Xiong, Qiuhong Bai, Cong Li, Huan Lei, Chaoyun Liu, Yehua Shen, Hiroshi Uyama

**Affiliations:** 1 Key Laboratory of Synthetic and Natural Functional Molecule Chemistry of Ministry of Education, College of Chemistry and Materials Science, Northwest University, Xi’an 710127, China; 92655927@163.com (Q.X.); baiqiuhong1990@163.com (Q.B.); licong716@163.com (C.L.); 2 College of Pharmaceutical Engineering, Shaanxi Fashion Engineering University, Xi’an 712046, China; 18192109687@163.com (H.L.); subwaycll@163.com (C.L.); 3 Department of Applied Chemistry, Graduate School of Engineering, Osaka University, Suita 565-0871, Japan

**Keywords:** cigarette filter, superhydrophobic, highly selective, absorbent, oil spillage

## Abstract

Ecological and environmental damage caused by oil spillage has attracted great attention. Used cigarette filters (CF) have also caused negative environmental consequences. Converting CF to economical materials is a feasible way to address these problems. In this study, we demonstrate a simple method for production of a highly hydrophobic absorbent from CF. CF was modified by using different volume ratios of octadecyltrichlorosilane and methyltrimethoxysilane. When the volume ratio was 3:2, the modified CF had the high water contact angle of 155°. It could selectively and completely absorb silicone oil from an oil-water mixture and showed a good absorption capacity of 38.3 *g*/*g*. The absorbed oil was readily and rapidly recovered by simple mechanical squeezing, and it could be reused immediately without any additional treatments. The as-obtained superhydrophobic modified CF retained an absorption capacity of 80% for pump oil and 82% for silicone oil after 10 cycles. The modified CF showed good elasticity in the test of repeated use. The present study provides novel design of a functional material for development of hydrophobic absorbents from used CF via a facile method toward oil spillage cleanup, as well as a new recycling method of CF to alleviate environmental impacts.

## 1. Introduction

Used cigarette filters (CF) are haphazardly discarded by people worldwide [1,2]. It is believed that there is 4.5 trillion CFs disposed into the environment every year all over the world [3]. Although the volume of a cigarette is small, it causes serious environmental effects [4]. Leaving a cigarette in a liter of water for four days has been reported to kill minnow fish species [5]. Extraction of nicotine, solanesol, and cellulose acetate from used CF has been examined [6,7], however the reported methods have some drawbacks such as long production cycles, high costs, and complicated procedures [8]. Thus, it is necessary to find a simple method to overcome the above shortcomings.

Water pollution caused by oil spills has attracted increasing attention due to increased quantity of industrial oily sewage as well as frequency of oil spill incidents [9,10,11]. For instance, the oil spill in Mexico at 2010 seriously threatened hundreds of fishes, birds and other biota living in the Gulf of Mexico. Great oil spills may destroy a whole ecological system and negatively affect economic activities. Various means are used to remedy these accidents including combustion, degradation and physical absorption of oils [12]. Among these methods, physical absorption via an absorbent is an effective method because of its low costs and lack of harmful by-products [10,13]. To remove spilled oils, different types of absorbents such as activated carbon [14,15], wool fibers [16] and zeolite have been developed [17]. However, they have disadvantages, for example, low absorption performance [18] and poor oil/water separation ability, which often restrict their usage [19]. Ultra-light carbonized materials from cellulose fibers show excellent oil capacity [20], but material reuse and oil recovery by means of combustion or distillation are arduous and inefficient processes [10].

Cellulose is the most abundant natural raw material in the world. Cellulose not only has good biodegradability and biocompatibility, but also has excellent thermal and chemical stability [21,22]. Many materials derived from fibers have been used for oil-water separation, for instance, cellulose polyesters [23,24], cellulose acetate [25,26], organogelator-cellulose [27], cellulose aerogels [28,29], cotton [30,31,32,33], paper [34] and CF [5,35]. Here, our goal is to transform CF into a superhydrophobic absorbent that can be repeatedly used for removal of spilled oils from water. CF is composed of cellulose acetate fibers with a porous structure, which is very important for absorbent materials [5].

Superhydrophobic fibers have recently attracted great interest because of their oil absorption features [36]. Wang et al. treated microcrystalline cellulose with hexadecyltrimethoxysilane through a sol–gel reaction [18]. Duan et al. treated chitin sponge with methyltrichlorosilane via a freeze-drying method [37]. Similarly, Liao et al. produced a cellulose aerogel modified with methyltrichlorosilane [38]. Many other celluloses were also used in oil-water separation by surface modification [39,40,41]. The conversion of hydroxyl groups on the fiber surface of cellulose acetate was monitored easily by spectroscopic analysis [26]. In the modification of cellulose acetate fibers by methyltrimethoxysilane, the alkoxysilane groups could condense on the fiber, leading to the superhydrophobic surface via chemical vapor deposition [12]. This structural modification facilitates applications of oil/water separation using fibrous materials modified by silane coupling agents; the hydrophobically modified fibers might ultimately exhibit oil/water separation characteristics. Obviously, the oil phase can spread and permeate easily through pores on the surface of the fibrous materials, while a water-impermeable barrier forms at the interface.

To the best of our knowledge, there are only two research groups that studied recycled CF as a superhydrophobic absorbent. In research by Ou et al., used CF was modified by hexadecyltrimethoxysilane to clean up oil spills on water [5]. Liu et al. reported a superhydrophobic and superoleophilic CF modified by octadecyltrichlorosilane for oil–water separation [35]. In this study, we report a simple and environmentally friendly method for preparation of superhydrophobic CF via methyltrimethoxysilane (MTMS) and octadecyltrichlorosilane (OTS) and the application for oil–water separation. OTS is a silane containing long carbon chain, which is favorable for increasing hydrophobicity. As is well known, MTMS is a cross-linking agent for cellulose [29]. The silylation level in the CF was adjusted by varying the initial MTMS concentration during the treatment [10,42]. In the present method, water drops could maintain a round shape with a contact angle greater than 150°, while oil drops quickly diffused on the surface. Microporous polymers, cross-linked polymer gels, macroporous gels and carbon-based foams were reported to efficiently remove oil from oil-water mixtures [23]. In comparison, the present CF evidently demonstrates economic effectiveness for oil-water separation. The absorbed oil could be readily recovered by simple mechanical squeezing, and the absorbent was immediately reused. Therefore, the present recycling of CF with its economic benefits and environmentally friendly characteristics will be a great approach for oil-water separation.

## 2. Experimental Section

### 2.1. Materials

Cigarette filters were collected from Central Japan Railway Company (Osaka, Japan). OTS, MTMS, toluene, pump oil, silicone oil, SiO_2_ nanoparticles with diameter of 10 nm and other organic reagents were purchased from Aladdin Industrial Corporation, Shanghai, China. All chemicals were used as received without further purification.

### 2.2. Fabrication of Hydrophobic SiO_2_ Particles

SiO_2_ (0.5 g) was ultrasonically dispersed in 20 mL of toluene, and 0.25 mL of OTS and 0.25 mL deionized water were added dropwise under stirring at ambient temperature. The mixture was stirred at ambient temperature for further 12 h. Finally, the resultant suspension was subjected to centrifugation and the obtained precipitate was separated and dried in an oven for 30 min at 80 °C.

### 2.3. Fabrication of Superhydrophobic Cigarette Filter

First, 0.5 g of the hydrophobic SiO_2_ was ultrasonically dispersed in 20 mL of toluene in the presence of 0.6 g CF under stirring at ambient temperature. The mixture was kept under magnetic stirring at 70 °C for 4 h after adding 0.25 mL of MTMS and 0.25 mL deionized water. Then, the reacted CF was placed in a vacuum oven for 72 h at 30 °C to remove the unreacted reagent and solvent. This product was recorded as CF_1:1_. Samples were similarly prepared from different feed ratios of OTS and MTMS and were recorded as CF*_x_*. For example, if the ratio of OTS to MTMS is 1 to 4, the product is denoted as CF_1:4_. A total of 7 samples, CF_1:0_, CF_4:1_, CF_3:2_, CF_1:1_, CF_2:3_, CF_1:4_ and CF_0:1_, were prepared according to the above method. These samples were stored in a desiccator for further use.

### 2.4. Characterization

Scanning electron microscopy (SEM) was used to observe the surface morphology of the samples. The samples for the SEM observation were first fixed on a sample holder using a carbon pad, and then, a thin gold film was formed on the samples by sputtering under vacuum. The SEM images were recorded on a HITACHI S-3000N instrument at 15 kV. Fourier transform infrared (FT-IR) spectra were recorded on a Vector 33 infrared spectroscope (Bruker Corporation, Karlsruhe, Germany). The scan range was 400–4000 cm^−1^. X-ray photoelectron spectroscopic (XPS) analysis was performed on a PHI 5000 VersaProbe II spectrometer (Physical electronics, Cambridge, MA, USA) with a monochromatic Al Kα source (1486 eV).

### 2.5. Wettability of Modified CF

Water contact angle (WCA) measurement was performed using an apparatus (OCA20) with a high-speed camera at ambient temperature. First, 4 μL of liquid was dropped automatically in a perpendicular way to the surface of the sample, and the contact angle was determined by using the fitting algorithm. The average value of the contact angle was obtained by measuring at five different positions of the sample surface. All the images were captured by a digital camera (Canon SX50 HS, Tokyo, Japan).

### 2.6. TGA Analyses

Thermogravimetric analysis (TGA) was conducted with a STA 449C thermogravimetric analyzer (NETZSCH, Selb, Germany) in the temperature range of 33 to 900 °C under a nitrogen atmosphere at 20 °C/min heating rate to demonstrate the availability of each components together with CF and sample analyses.

### 2.7. Evaluation of Sorption Capacities of Modified CF for Oils or Organic Solvents

The adsorption capacity of oil (or organic solvent) is defined as the quality of the absorbed oil (g) per unit mass of dry absorbent (g), which is used as a measure of the oil absorption capacity. A weighed hydrophobic cigarette filter (CF) was placed in an oil or organic solvent for 1 min to reach its absorption equilibrium. It was then drained for several seconds and removed for the weight measurement. For each sample, the weight was measured at least three times, and the average value was presented.

### 2.8. Recyclability of Modified CF

The absorbed oil or organic solvent in the modified CF was recovered by simple mechanical extrusion. Then, the oil inside the modified CF was squeezed out without any additional treatments, and the weight of the CF before and after the squeeze process was measured. The oil storage capacity, which is defined as the mass of oil (g) per unit mass of the dry CF (g), is used to evaluate the recyclability of the modified CF.

### 2.9. Evaluation of Oil Sorption Selectivity of Modified CF

The process to absorb oil by the modified CF was described in 2.7. For visual observation of the selective oil sorption, the oil-absorbed modified CF was placed in a beaker and the top of the beaker was wrapped by the film. Then, the beaker was dried by hot air at 105 °C for 5 min. An empty beaker was also dried under the same conditions as a control experiment. The separation efficiency of oil and water was evaluated by observing whether water droplets were found in the film and the inner wall of the beaker.

### 2.10. Qualitative Analysis of Oil-water Separation Efficiency

A mixture of silicone oil and 0.1 M sodium chloride (NaCl) solution (dyed with methyl violet) was prepared to evaluate the quantitative analysis of the oil-water separation efficiency. A silver nitrate (AgNO_3_) aqueous (0.1 M) solution was dyed with methyl violet in the test beaker. The as-prepared oil-water mixture was then poured into a vial through the hydrophobic CF_3:2_. Because of its high sensitivity to Cl^−^ ion, AgNO_3_ is reacted with NaCl to immediately form a whitish AgCl precipitate. Therefore, even a minute amount of NaCl in the penetrate through the hydrophobic CF_3:2_ would be detected. By observing the formation of silver chloride precipitation, the separation efficiency is qualitatively evaluated. Note that oil is lighter than water, so water was added immediately, followed by oil.

## 3. Results and Discussion

### 3.1. Fabrication of Superhydrophobic Cigarette Filters

**Figure 1:**
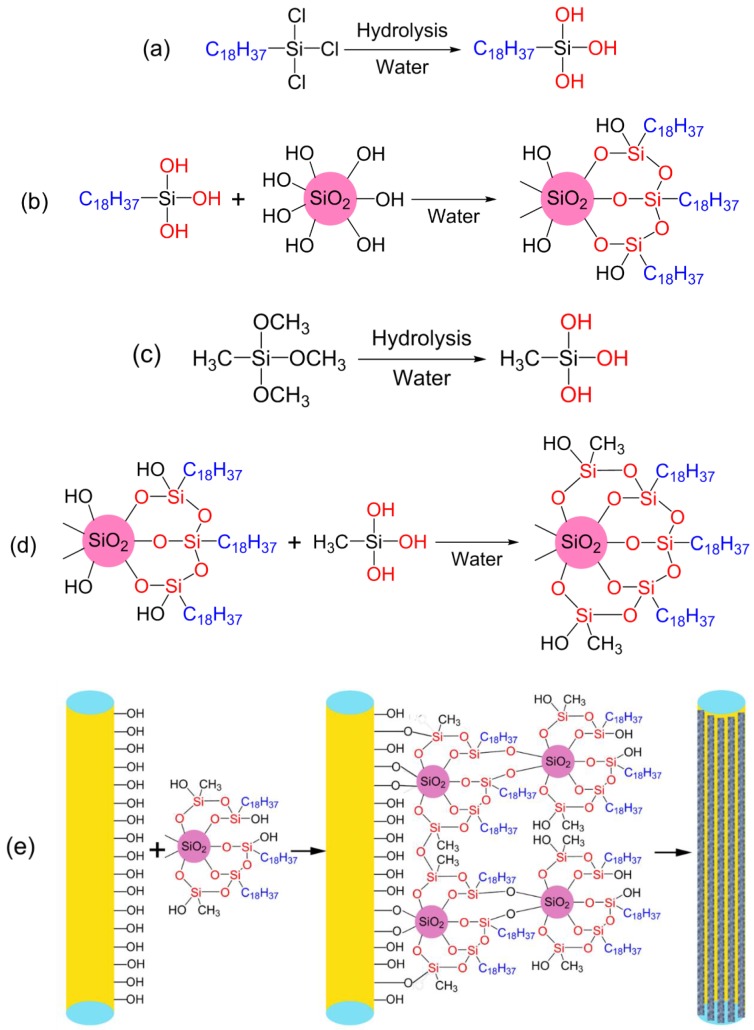
Fabrication processes of a superhydrophobic absorbent from CF. Hydrolysis of OTS (**a**); fabrication of hydrophobic SiO_2_ particles (**b**); hydrolysis of MTMS (**c**); fabrication of hydrophobic SiO_2_@OTS@MTMS particles (**d**); and fabrication of superhydrophobic CF (**e**).

Preparation scheme of the hydrophobic CF is schematically presented in Figure 1. SiO_2_ particles have many silanol (SiOH) groups on the surface, which form aggregates during the drying step; therefore, SiO_2_ nanoparticles are an ideal raw material for the fabrication of organic/inorganic hybrid materials [43,44]. The addition of SiO_2_ strongly enhances the flux [45,46] which promotes the adhesion of OTS onto the CF surface. Hydrolysis of OTS occurs in water, and each molecule of OTS newly generates 3 silanol groups (Figure 1a). This silanol is bound to the surface of SiO_2_. The SiO_2_@OTS particles were formed by mixing SiO_2_ nanoparticles and OTS (Figure 1b). The flexible interlayer of SiO_2_ reduces the surface tension and stress concentration to prevent chipping or crack during operations [43]. Furthermore, the SiO_2_ particles provide roughness for preparing superhydrophobic layers. OTS also reduced the surface energy. The SiO_2_@OTS particles would have the mechanical properties of the hydrophobic layer, and the hydrophobic layer demonstrates better stability and durability.

A small amount of water induced the hydrolysis of MTMS, to generate 3 silanol groups (Figure 1c). The resulting silanol groups would be reacted either with remaining silanol groups of the SiO_2_@OTS particles or the hydroxyl groups on the surface of the modified CF. Once MTMS was immersed in the dispersion of the SiO_2_@OTS particles in toluene, both are bonded together by Si-O-Si bonds (Figure 1d). The modified CF quickly absorbs the SiO_2_@OTS@MTMS particles and individual OTS and MTMS molecules to afford the hydrophobic CF in the cost-effective and environmentally friendly processes. The silanol groups are reacted with the hydroxyl groups of CF to form covalently bonded layers on the surface of the modified CF (Figure 1e). By these reaction processes, CF are uniformly coated by the layers of the SiO_2_@OTS@MTMS particles.

### 3.2. Surface Morphology and Characterization of Modified Cigarette Filters

**Figure 2:**
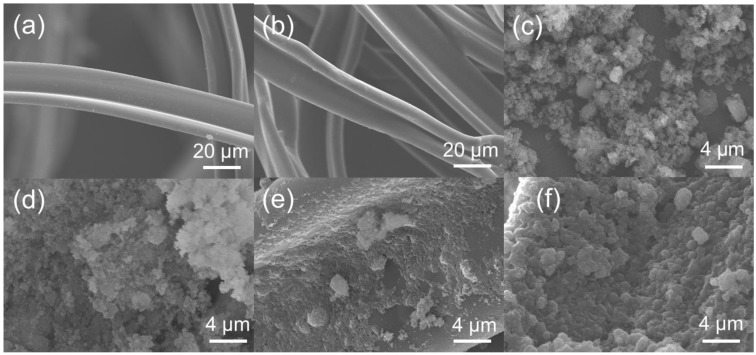
SEM images of new cigarette filter (**a**), used cigarette filter (**b**), SiO_2_ particles (**c**), SiO_2_@OTS (**d**), CF modified by SiO_2_@OTS (**e**), and CF modified by SiO_2_@OTS@MTMS (**f**).

To fabricate the superhydrophobic CF, the modification with MTMS and OTS was examined. The morphology of the CF before and after the modification are shown in Figure 2. New and used CF have similar morphology with the smooth surface (Figure 2a,b). SEM images of SiO_2_ and SiO_2_@OTS particles are shown in Figure 2c,d, respectively, suggesting the small morphological change of the SiO_2_ particles. After the modification by SiO_2_@OTS, the fiber surface was not smooth and the particles were not completely covered on the surface (Figure 2e and Figure S3c,d). The formation of the polysiloxane particles is attributed to the self-polymerization of silanols. With the further modification by MTMS, the surface became increasingly rougher with wrinkles (Figure 2f and Figure S3e,f). SEM images of unmodified CF and modified CF_1:0_, CF_4:1_, CF_3:2_, CF_1:1_, CF_2:3_, CF_1:4_, and CF_0:1_ are shown in Figure S1. The three-dimensional reticular layer by the continuous integration of the OTS or MTMS moiety on the SiO_2_ particles as core was observed (Figure 2f and Figure S2). The morphological change in macroporous and microscopic structures before and after modification can be seen more clearly by the SEM images in different magnification (Figure S3). The generation of this coating layer is ascribed to the hydrolysis of OTS and MTMS to form the hydroxyl groups and the subsequent assembly on the surface of the cellulose acetate fiber. The assembled coating layer along with the inherent rough texture of CF could lead to the hydrophobic character of the surface. There are the peaks attributed to the silicon atom in the EDX spectra of the resulting CF samples (Figure S4). In addition, the silicon element is evenly distributed across the surface of the modified CF, whereas the peak due to silicon did not appear in CF without the modification, confirming the successful silanization reaction on the surface of CF. The results given by SEM were consistent with the previous reaction mechanism.

**Figure 3:**
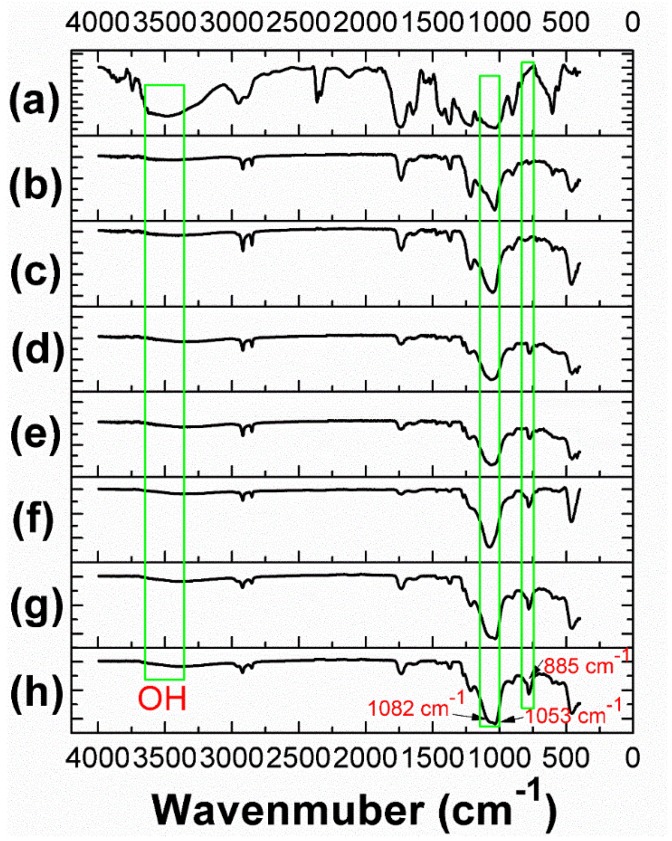
FTIR spectra of unmodified and modified CF: CF_0:0_ (**a**), CF_1:0_ (**b**), CF_4:1_ (**c**), CF_3:2_ (**d**), CF_1:1_ (**e**), CF_2:3_ (**f**), CF_1:4_ (**g**), and CF_0:1_ (**h**).

Figure 3 shows the FTIR spectra of pristine CF and SiO_2_@OTS@MTMS-modified CF samples. A broad peak at 3440 cm^−1^ is assigned to the O-H group and the weakening for the modified CF samples is clearly observed, indicating that the hydroxyl group on the surface of the cellulose acetate fibers were incorporated into SiO_2_, OTS, MTMS, SiO_2_@OTS, or SiO_2_@OTS@MTMS particles. The decrease of the peak intensity of the hydroxyl group is strongly related to the enhanced hydrophobicity of the modified CF. The three-dimensional network structure formed by the SiO_2_@OTS@MTMS particles also enhanced the hydrophobicity of the modified CF. After the modification by the SiO_2_@OTS@MTMS particles at different volume ratios (1:0, 4:1, 3:2. 1:1, 2:3, 1:4, 0:1), observed are new peaks around 1082 and 1053 cm^−1^ due to the Si–O stretching and bending vibrations, respectively [5]. The detection of Si–O implies that the silanol group from OTS and MTMS was reacted with the hydroxyl group of cellulose acetate. The peak around 885 cm^−1^ belongs to the Si–OH vibration, which indicates that the hydrolysis of OTS or MTMS occurred. These data were also consistent with the EDX results; the strong peaks ascribed to C, O, and Si were found in the EDX spectrum (Figure S4), confirming that the surface of the SiO_2_@OTS@MTMS-modified CF were mainly composed of C, O, and Si.

XPS analysis was made to investigate the surface chemistry and bonding status of the modified CF, confirming the presence of C, O, and silicon atoms in the samples (Figure S5). According to the XPS survey results, peaks due to silicon appeared at 101.6 and 154.2 eV for Si 2p and Si 2s, respectively. A high-resolution XPS spectrum for the Si 2p core is presented in Figure S5b. These peaks are obviously absent in the pristine CF survey spectrum. The CF modified only by OTS or MTMS also had the similar peaks. Consequently, the XPS analysis clearly indicate the presence of silicon atoms on the surface of the modified CF and the obtained results well agrees with those by the FTIR and EDX analysis. The silanol group formed by the hydrolysis of OTS or MTMS is combined solely with the hydroxyl group on the surface of the cellulose acetate fibers, which enhanced the hydrophobicity of CF as described later. To further discuss the effect of modification for different volume ratios of OTS to MTMS on CF, the hydrophobicity of the modified CF was analyzed in the next section.

### 3.3. Hydrophobicity of SiO_2_@OTS@MTMS-modified Cigarette Filters

**Figure 4:**
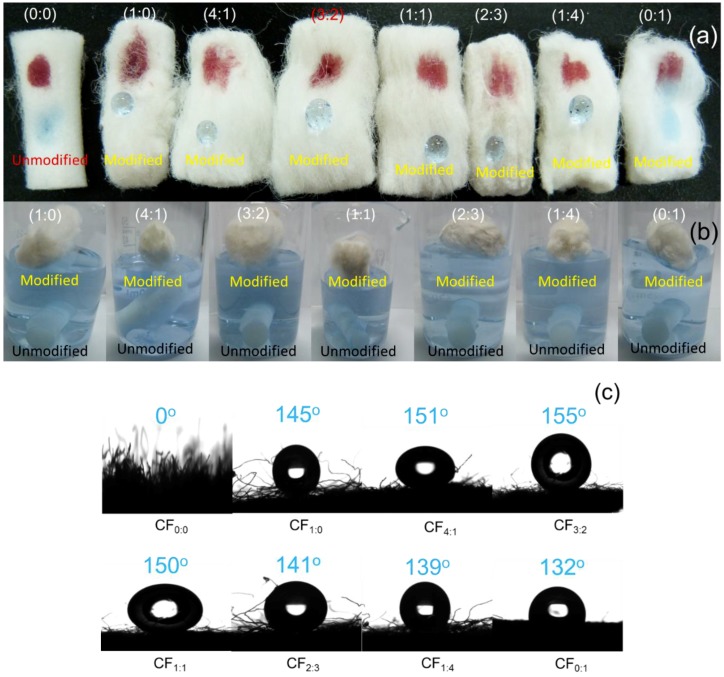
Optical images of water and oil droplets on unmodified and modified CF (**a**), soaking in water (**b**) and water contact angle (**c**). Droplets of water (blue) and silicone oil (red) were colored with bromophenol blue and Sultan three, respectively.

Evident changes of the wettability of the hydrophobic CF was found in the process of preparing SiO_2_@OTS@MTMS-modified CF. When the unmodified CF and modified hydrophobic CF were placed in water simultaneously, the unmodified sample rapidly sank into water, while the hydrophobic sample floated well on the water surface (Figure 4a). When CF_3:2_ was immersed in the water, it floated from the bottom of the water to the top (Video S1). This highly hydrophobic sample could not be wetted, and no water was absorbed. To further verify its hydrophobicity, CF_3:2_ was placed on a ruler with an angle of 45 degree between the ruler and a beaker. When the water dropped down, the water droplet flowed away quickly from CF_3:2_ (Video, S2) indicating that the SiO_2_@OTS@MTMS-modified CF showed high hydrophobicity.

Because of amphiphilic property of cellulose acetate, water and oil droplets can be rapidly adsorbed onto the unmodified CF, suggesting that the separation of oil and water cannot be carried out effectively by using the unmodified CF. On the other hand, water droplets remained on the surface of the modified CF, while a pump oil was absorbed immediately, indicating the lipophilic and hydrophobic characteristics of the modified CF (Figure 4a). All the CF samples modified by different volume ratios of OTS to MTMS floated on the water surface, indicating that the modified CF showed good hydrophobicity. On the other hand, the water droplet could not stand on the surface of the CF modified only by MTMS (CF_0:1_) (Figure 4b). These results indicate the lower hydrophobicity of CF_0:1_ than that of other modified CF samples.

Effective oil/water selectivity was necessary for oil absorbents. Because of the large quantity of hydrophilic hydroxyl groups in cellulose acetate, CF cannot be directly used as oil absorption materials. As shown in Figure 4c, a water droplet quickly penetrated the unmodified CF. However, a water droplet could stand on the surface of the modified CF and sufficiently keep its original shape. WCA values of CF_1:0_, CF_4:1_, CF_3:2_, CF_1:1_, CF_2:3_, CF_1:4_, and CF_0:1_ with different volume ratio of OTS to MTMS were 145°, 151°, 155°, 150°, 141°, 139° and 132°, respectively, indicating their hydrophobic feature. Note that WCA value of some samples was higher than 150°, indicating the superhydrophibic property of the present modified CF. There is no clear tendency of the WCA value for these samples; WCA of CF_3:2_ was slightly larger than that of other samples. It may be because there was a large number of longer alkyl chains derived from OTS on the fiber surface, which was more hydrophobic than those from MTMS. WCA of CF_4:1_ was lower than that of CF_3:2_.

For investigating the thermal character, the durability of the CF and CF_3:2_ were tested using TGA, as shown in Figure S6. Compared with the CF, the TGA curves of modified CF_3:2_ exhibit a certain difference. It showed that early decomposition stages (250−320 °C) for the attached SiO_2_@OTS@MTMS part were easily noticeable. About 92% of the total material weight has been removed until 900 °C and there were about 18% hydrophobic functional group in modified CF_3:2_.

### 3.4. Elasticity Property of Modified Cigarette Filters

**Figure 5:**
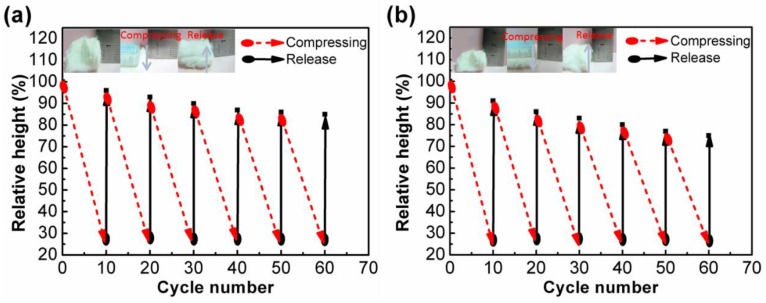
Height recovery of hydrophobic CF_3:2_ (**a**) and CF_4:1_ (**b**).

As shown in Figure 5, the ruler quickly compressed the modified CF_3:2_ to one-fifth of its original height. The movement was repeated 60 times, and the change in height was constantly observed. After the 60 cycles, CF_3:2_ recovered to almost 85% of its original height. In the similar experiment for CF_4:1_, the recovery ratio in height was 74%. These results indicate the good elasticity of CF_3:2_. The excellent elasticity would greatly facilitate the oil recovery and reuse of the CF_3:2_.

### 3.5. Oil Selectivity Absorption

**Figure 6:**
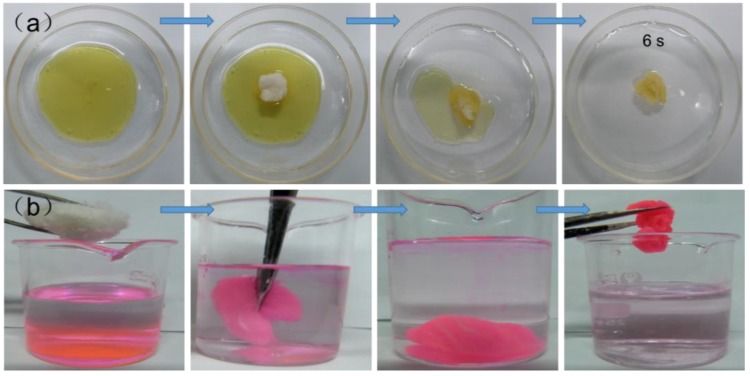
Absorption of pump oil (**a**) and chloroform (**b**) by CF_3:2_. Chloroform was colored with Rhodamine B.

The unmodified CF is easily wetted by water. When CF_3:2_ was placed in a mixture of pump oil and water, it floated completely on the surface of water and only absorbed pump oil, showing high selectivity (Figure 6a). CF_3:2_ floated on the water without releasing the absorbed pump oil, supporting good performance for the separation of oil and water. Within a very short period (6 s), CF_3:2_ completely absorbed the pump oil (Video, S3) due to its high hydrophobicity. This highly rapid absorption would satisfy the requirements in real applications of oil/water separation. In addition, CF_3:2_ could also easily absorb chloroform from the bottom of the water within a few sec, implying that CF_3:2_ showed good absorption property both for oil and organic solvents (Figure 6b).

To verify the oil sorption selectivity of the modified CF, three kinds of beakers, a beaker with the pump oil-absorbed CF_3:2_ (see experimental), one with a small amount of water, and an empty beaker, were prepared, dried in air at 105 °C for 5 min, and then cooled to room temperature for 30 s (Figure S7). The experiments using the latter two beakers were conducted as reference. The top of all the beakers was covered by the film. In case of the beaker with water, the condensed water was observed in the film and the inner wall of the beaker; however, such water condensation was not found for CF_3:2_ and empty beaker (Figure S7). These results clearly demonstrate that CF_3:2_ only absorbed pump oil; water was not contained in the absorbed oil, confirming the high absorption selectivity between water and oil.

**Figure 7:**
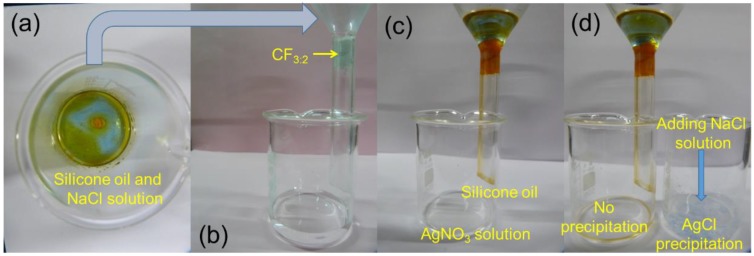
Separation efficiency test of oil absorption by CF_3:2_.

The above qualitative test may not be accurate for the oil samples containing a very small amount of water, which is often difficult to detect. Thus, another method to detect a small amount of water was examined for the demonstration of the excellent separation efficiency of CF_3:2_. (Figure 7). It is well known that Ag^+^ is very sensitive to Cl^−^. Even if a small amount of Ag^+^ was in contact with Cl^−^, a precipitate of AgCl would form. A silver nitrate (AgNO_3_) aqueous (0.1 M) solution was placed in the test beaker. When a mixture of NaCl aqueous solution (0.1 M) and silicone oil was poured in a funnel bunged with CF_3:2_, the silicone oil penetrated through CF_3:2_ and dropped into the test beaker beneath it (Figure 7a,b). Meanwhile, water was not penetrated into CF_3:2_. The AgNO_3_ aqueous solution in the test beaker remained as clear as the initial condition after the oil/water separation (Figure 7c), which clearly supports the high separation efficiency and high hydrophobicity (Figure 7d). In the control experiment, a precipitate was generated. The excellent wettability of the modified CF_3:2_ toward oils make it promising as a material for oil/water separation. Thus, the as-prepared superhydrophobic CF_3:2_ is a good candidate for industrial applications such as oil-polluted water treatment and oil spill cleanup.

### 3.6. Reusability

**Figure 8:**
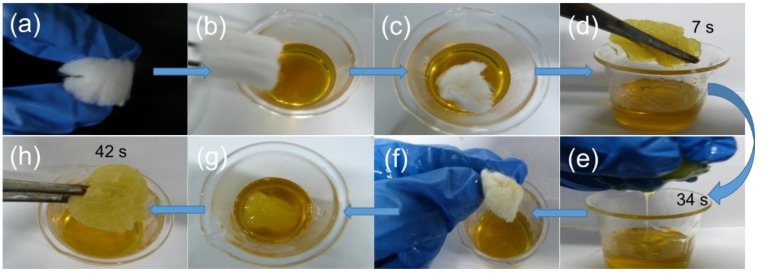
Pump oil recovery and reuse of CF_3:2_.

As shown in Figure 8, CF_3:2_ could be saturated with pump oil only in 7 s (Figure 8a–d), exhibiting high absorption efficiency. More importantly, CF_3:2_ could be reused for oil/water separation, and the absorbed pump oil could be readily recovered by simple mechanical squeezing (Figure 8e–f). Furthermore, the color of CF_3:2_ changed back to white, indicating that the pump oil was quantitatively squeezed from CF_3:2_. Interestingly, the squeezed CF_3:2_ could quickly absorb pump oil again (Figure 8g), and it almost recovered its volume without any post treatments (Figure 8h), demonstrating its high efficiency in oil recovery and excellent reusability. The process was recorded by a video camera (Video, S4). It took only 7 s to absorb the pump oil and 27 s to squeeze out the oil. The whole cycle took no more than 45 s, showing the high efficiency for oil absorption. Other recycling methods used in oil recovery such as distillation, solvent extraction, and combustion, usually involve complex and time consuming processes, especially for carbon-based absorbents; they often show brittle nature, which makes their reuse and oil recovery less efficient [10]. As shown above, CF_3:2_ easily recovered the oil, and was effectively utilized through simple compression, which is very important for practical applications.

### 3.7. Oil Adsorption Capacities of Different Hydrophobic Cigarette Filters

**Figure 9:**
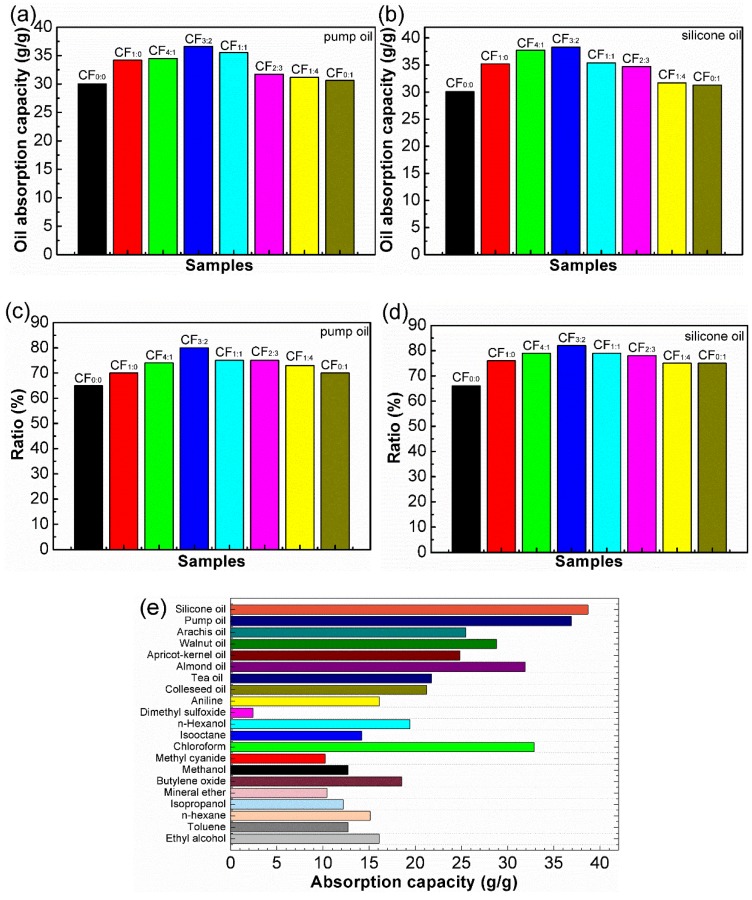
Absorption capacities of unmodified and modified CF (**a**,**b**), 10 times usage (**c**,**d**) and CF_3:2_ for various oils, organic solvents (**e**).

As shown in Figure 9a,b, the oil absorption capacity of different hydrophobic CF samples was investigated. The porous and interconnected skeleton of the hydrophobic CF provided large volume for oil storage [45]. The oil absorbency for most of the reported highly hydrophobic materials was approximately 10 times their own weight [45]. Our developed materials, CF_1:0_, CF_4:1_, CF_3:2_, CF_1:1_, CF_2:3_, CF_1:4_ and CF_0:1_, had the absorption capacity for pump oil of 34.2 *g*/*g*, 34.5 *g*/*g*, 36.6 *g*/*g*, 35.5 *g*/*g*, 31.7 *g*/*g*, 31.2 *g*/*g*, and 30.6 *g*/*g*, and for silicone oil of 35.2 *g*/*g*, 37.7 *g*/*g*, 38.3 *g*/*g*, 35.4 *g*/*g*, 34.7 *g*/*g*, 31.7 *g*/*g*, and 31.3 *g*/*g*, respectively. These data were greatly superior to most of the reported values. The capacity of the hydrophobic CF was larger than that of the unmodified CF for pump and silicone oils. A similar tendency of the absorption capacity of the samples of different volume ratios of OTS to MTMS for both oils was found, and CF_3:2_ had the largest capacity toward both oils. This modification obviously enhanced the hydrophobicity and the absorption of oils or organic solvents.

To further verify the reusability capacity of the modified CF, 10 times absorption of pump and silicone oils was performed. The absorption rate decreased slowly with multiple cycles of the absorption (Figures S8 and S9). After 10 cycles, CF_3:2_ maintained an absorption capacity of maximum 80% for pump oil and 82% for silicone oil, demonstrating the good stability of the present materials (Figure 9c,d). The unmodified CF also retained an absorption capacity of 65% for pump oil and 66% for silicone oil. These data show that the modified hydrophobic CF had better reusability capacity than the unmodified CF due to its excellent elasticity. These results agree with the elasticity and reusability analysis as described above.

Frequently encountered organic liquids in daily life and industry, food oils, alkane, chloroalkanes, alcohol, and aromatic compounds, were used to evaluate the absorbency of CF_3:2_. Figure 9e shows the absorption capacity of CF_3:2_ for such liquids. CF_3:2_ showed larger oil absorption capacity than organic solvents. The absorption capacities of silicone oil, pump oil, arachis oil, walnut oil, apricot kernel oil, almond oil, tea oil, and colleseed oil were 38.3 *g*/*g*, 36.6 *g*/*g*, 25.5 *g*/*g*, 28.8 *g*/*g*, 24.8 *g*/*g*, 31.9 *g*/*g*, 21.8 *g*/*g*, and 21.2 *g*/*g*, respectively, and the absorption capacities for aniline, dimethyl sulfoxide, n-hexanol, isooctane, methyl cyanide, methanol, butylene oxide, mineral ether, isopropanol, n-hexane, toluene and ethanol were 16.1 *g*/*g*, 2.4 *g*/*g*, 19.4 *g*/*g*, 14.2 *g*/*g*, 32.9 *g*/*g*, 10.2 *g*/*g*, 12.7 *g*/*g*, 18.5 *g*/*g*, 10.4 *g*/*g*, 12.2 *g*/*g*, 15.1 *g*/*g*, 12.7 *g*/*g*, and 16.1 *g*/*g*, respectively. Interestingly, the absorption capacity for chloroform (32.9 *g*/*g*) was the largest among the organic solvents examined. The absorption variation depends on density of an oil or organic solvent [28] and the surface tension and viscosity of organics also influence oil absorbency [45], which would explain the reason why the hydrophobic CF had greater capacity for oils than organic solvents.

Cellulose acetate filters were attached to cigarettes in the 1950s, to reduce the yield of tar and nicotine when smoking [8]. However, used CF degrades very slowly and thus represent an accumulating mass of potentially toxic waste [47]. Few scholars studied recycling of CF [6,7,48,49,50,51,52,53,54] (Table S1). Previous studies on CF recycling have mainly focused on use of CF as an absorbent or carbon source. In this study, on the other hand, CF was applied to oil and water separation not only to reduce environmental pollution but also to address oil spill cleanup and chemical leakage. As shown in Table 1 [55,56,57], the methods for oil recovery and absorbent recycling such as solvent extraction, vacuum filtration, pump extrusion, freeze-drying, and heating are usually complicated, time consuming and energy intensive, and have low efficiency for most absorbents. Compared with reported recycling methods for absorbents, squeezing was the most facile and cost-effective. For the fibers used for the oil recovery, involved were two or three steps such as squeezing and solvent extraction, compression, rinsing and freeze-drying. In this work, the absorbed oil could be recovered by facile mechanical squeezing without a combination of other methods. Furthermore, highly hydrophobic, cost-effective, and environmentally friendly CF retained high absorption capacity after 10 cycles. The structure and elasticity also had no obvious damage in repeated reuse.

## 4. Conclusions

This study provides a facile method for the preparation of the modified CF via SiO_2_, OTS and MTMS and the application of oil and water separation. The modified CF exhibited high hydrophobicity by the surface modification with different volume ratios of OTS to MTMS. CF_3:2_ had the largest WCA of 155° and could selectively absorb silicone oil from an oil-water mixture. This superhydrophobic absorbent had good elasticity and exhibited high reusable capacity for oil absorption. Furthermore, the absorbed oil could be readily and rapidly recovered by simple mechanical squeezing without any additional treatments. The oil recovery and recycling method, recycling efficiency, cost, oil selectivity, elasticity, environmental friendliness and hydrophobicity of the present CF were superior to those of reported fiber absorbents. The modified CF could be applied to oil and water separation not only to reduce environmental pollution, but also to address oil spill cleanup and chemical leakage.

## Figures and Tables

**Table 1:** Comparison of various fiber absorbents developed in recent years ^a^.

Time	Materials	Oil Recovery and Reuse Method	Reuse Efficiency	Cost	Environment Friendly	Ref
2014	polyester fabric	squeezing and solvent extraction	XX	+		[46]
2014	nanofibrillated cellulose	solvent extraction	X	+	Yes	[34]
2014	cotton	vacuum filtration	X	+	Yes	[27]
2015	polyurethane	extrusion by pump	√	+		[32]
2015	cellulose fiber	squeezing	√√	−−		[9]
2015	bacterial cellulose	compression, rinsing and freeze-drying	XX	++		[25]
2015	cigarette filters	extrusion and washed with ethanol	√	−	Yes	[28]
2016	polypropylene	squeezing	√√	−		[47]
2017	ethyl cellulose	heating	√	−		[48]
2018	cigarette filters	squeezing	√√	−−	Yes	This work

^a^ Note: “X” complicated, “XX” very complicated, “√” facile, “√√” very facile, “−” low, “−−” very low, “+” high, “++” very high.

## Supplementary Materials

The following are available online at http://www.mdpi.com/2073-4360/10/10/1101/s1, Figure S1: SEM images of unmodified and modified CF: CF_0:0_ (a), CF_1:0_ (b), CF_4:1_ (c), CF_3:2_ (d), CF_1:1_ (e), CF_2:3_ (f), CF_1:4_ (g), and CF_0:1_ (h), Figure S2: SEM images of SiO_2_ particles (a) and (b) at different magnification, SiO_2_/OTS (c), and SiO_2_/OTS/MTMS (d), Figure S3: SEM images of CF modified by SiO_2_/OTS (a and b), and CF modified by SiO_2_/OTS/MTMS (c and d) at different magnification, Figure S4: EDX spectra of unmodified and modified CF: CF_0:0_ (a), CF_1:0_ (b), CF_4:1_ (c), CF_3:2_ (d), CF_1:1_ (e), CF_2:3_ (f), CF_1:4_ (g), and CF_0:1_ (h), Figure S5: Wide scan of XPS spectra of unmodified and modified CF (a) and high-resolution spectrum for Si 2p of CF_1:1_ (b), Figure S6: TGA thermograms of the CF and CF_3:2_Selectivity test of oil absorption by CF_3:2_, Figure S7: Selectivity test of oil absorption by CF_3:2_, Figure S8: Pump oil absorption of unmodified and modified CF: CF_0:0_ (a), CF_1:0_ (b), CF_4:1_ (c), CF_3:2_ (d), CF_1:1_ (e), CF_2:3_ (f), CF_1:4_ (g), and CF_0:1_ (h), Figure S9: Silicone oil absorption of unmodified and modified CF: CF_0:0_ (a), CF_1:0_ (b), CF_4:1_ (c), CF_3:2_ (d), CF_1:1_ (e), CF_2:3_ (f), CF_1:4_ (g), and CF_0:1_ (h), Table S1: Recycling of used cigarette filters, Video S1: CF_3:2_ floated from the bottom of the water to the top, Video S2: Water droplet flowed away quickly from CF_3:2_, Video S3: CF_3:2_ had completely adsorbed the pump oil with 6 s, Video S4: CF_3:2_ was reused, and the absorbed pump oil could be readily recovered by simple mechanical squeezing.
