# Supplementary material for: Cost-Effective, Highly Selective and Environmentally Friendly Superhydrophobic Absorbent from Cigarette Filters for Oil Spillage Clean up
Source: Polymers (Basel). 2018 Oct 5;10(10):1101. doi: 10.3390/polym10101101 (PMC6403843; doi:10.3390/polym10101101)

Supporting Information

Cost-effective, Highly Selective and Environmentally Friendly Superhydrophobic Absorbent from Cigarette Filters for Oil Spillage Cleanup

Qiancheng Xiong^a,b^, Qiuhong Bai^a^, Cong Li^a^, Huan Lei^b^, Chaoyun Liu^b^, Yehua Shen^a,^*, Hiroshi Uyama^a,c,^*

^a^Key Laboratory of Synthetic and Natural Functional Molecule Chemistry of Ministry of Education, College of Chemistry and Materials Science, Northwest University, Xi’an 710127, Shaanxi Province, China

^b^College of Pharmaceutical Engineering, Shaanxi Fashion Engineering University, Xi’an 712046, Shaanxi Province, China

^c^Department of Applied Chemistry, Graduate School of Engineering, Osaka University, Suita 565-0871, Japan

*Corresponding author: Tel: +86-29-88302635; E-mail: yhshen@nwu.edu.cn

*Corresponding author. Tel.: +81-6-6879-7364; Fax: +81-6-6879-7367;

E-mail: [uyama@chem.eng.osaka-u.ac.jp](mailto:uyama@chem.eng.osaka-u.ac.jp)

**Content:**

Figure S1. SEM images of unmodified and modified CF: CF_0:0_ (a), CF_1:0_ (b), CF_4:1_ (c), CF_3:2_ (d), CF_1:1_ (e), CF_2:3_ (f), CF_1:4_ (g), and CF_0:1_ (h).

Figure S2. SEM images of SiO_2_ particles (a) and (b) at different magnification, SiO_2_/OTS (c), and SiO_2_/OTS/MTMS (d).

Figure S3. SEM images of CF modified by SiO_2_/OTS (a and b), and CF modified by SiO_2_/OTS/MTMS (c and d) at different magnification.

Figure S4. EDX spectra of unmodified and modified CF: CF_0:0_ (a), CF_1:0_ (b), CF_4:1_ (c), CF_3:2_ (d), CF_1:1_ (e), CF_2:3_ (f), CF_1:4_ (g), and CF_0:1_ (h).

Figure S5. Wide scan of XPS spectra of unmodified and modified CF (a) and high-resolution spectrum for Si 2p of CF_1:1_ (b).

Figure S6. TGA thermograms of the CF and CF_3:2_

Figure S7. Selectivity test of oil absorption by CF_3:2_.

Figure S8. Pump oil absorption of unmodified and modified CF: CF_0:0_ (a), CF_1:0_ (b), CF_4:1_ (c), CF_3:2_ (d), CF_1:1_ (e), CF_2:3_ (f), CF_1:4_ (g), and CF_0:1_ (h).

Figure S9. Silicone oil absorption of unmodified and modified CF: CF_0:0_ (a), CF_1:0_ (b), CF_4:1_ (c), CF_3:2_ (d), CF_1:1_ (e), CF_2:3_ (f), CF_1:4_ (g), and CF_0:1_ (h).

Table S1. Recycling of used cigarette filters

Video S1. CF_3:2_ floated from the bottom of the water to the top.

Video S2. Water droplet flowed away quickly from CF_3:2_.

Video S3. CF_3:2_ had completely adsorbed the pump oil with 6 s.

Video S4. CF_3:2_ was reused, and the absorbed pump oil could be readily recovered by simple mechanical squeezing.

20 µm

(a)

(b)

**
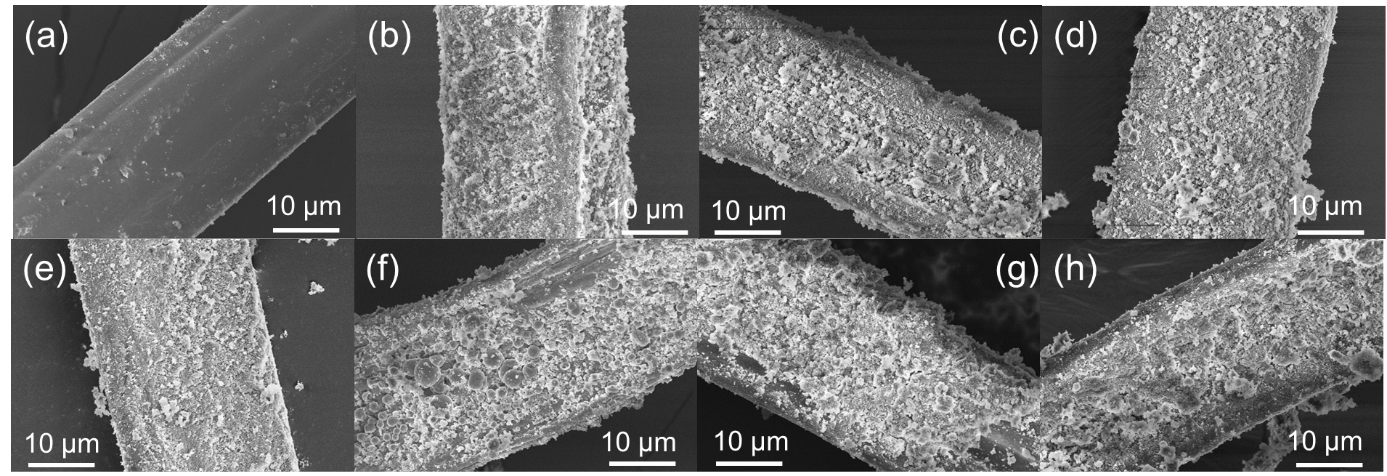
**

Figure S1. SEM images of unmodified and modified CF: CF_0:0_ (a), CF_1:0_ (b), CF_4:1_ (c), CF_3:2_ (d), CF_1:1_ (e), CF_2:3_ (f), CF_1:4_ (g), and CF_0:1_ (h).


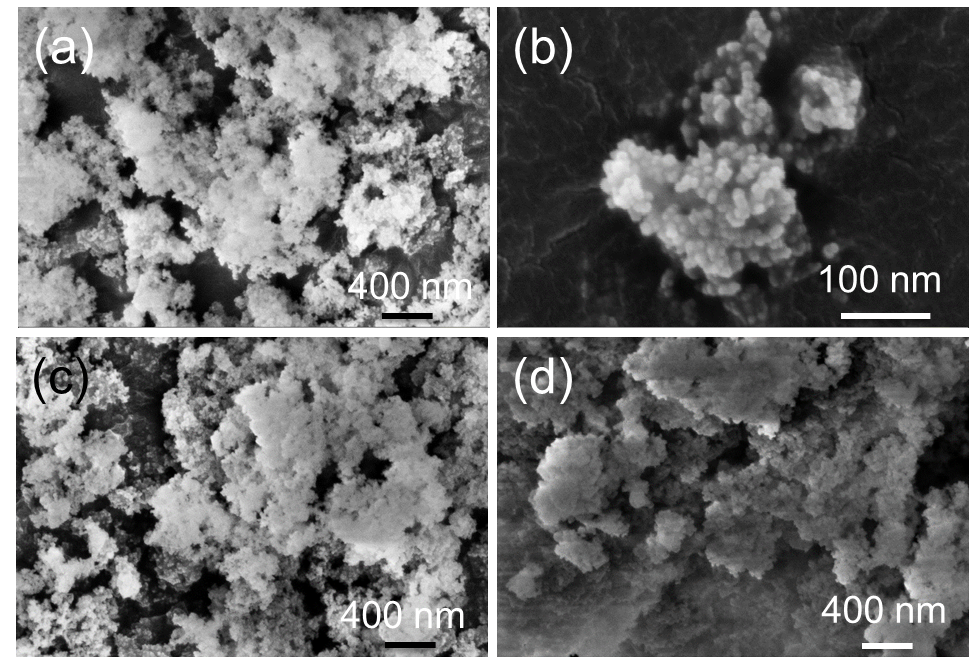


Figure S2. SEM images of SiO_2_ particles (a) and (b) at different magnification, SiO_2_/OTS (c), and SiO_2_/OTS/MTMS (d).


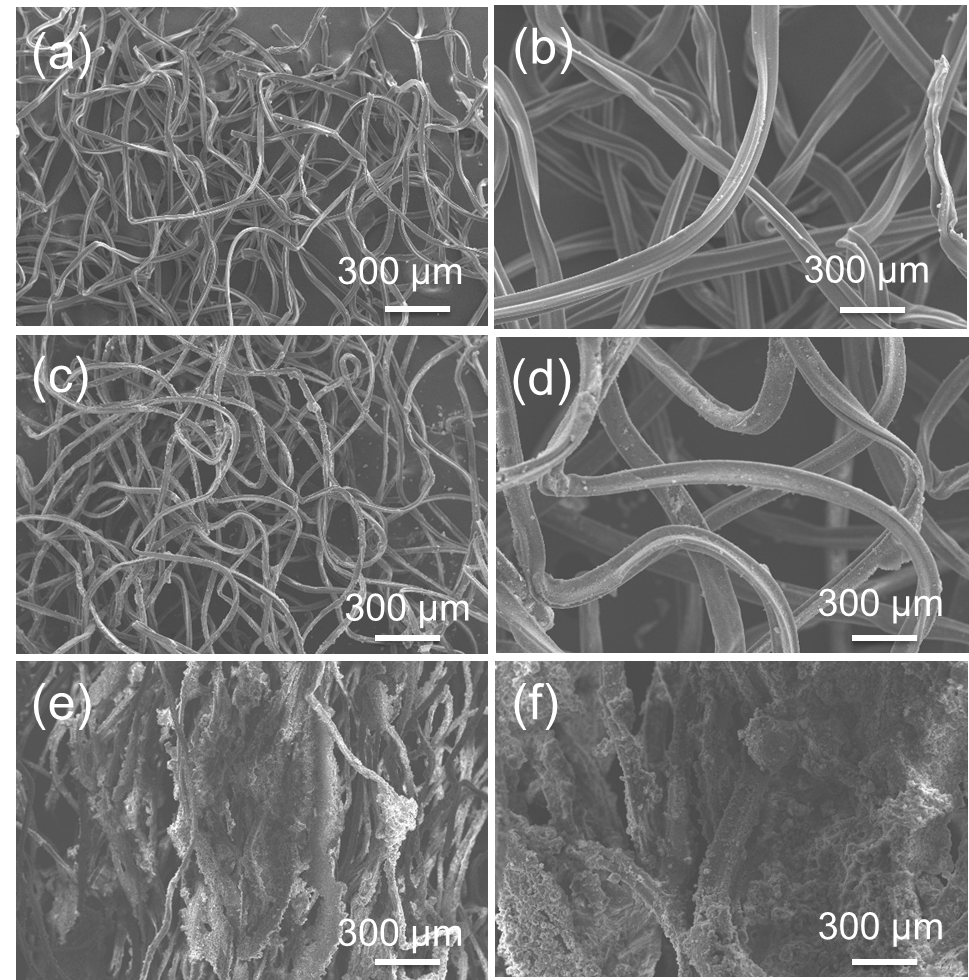


Figure S3. SEM images of CF (a and b), CF modified by SiO_2_/OTS (a and b), and CF modified by SiO_2_/OTS/MTMS (c and d) at different magnification.


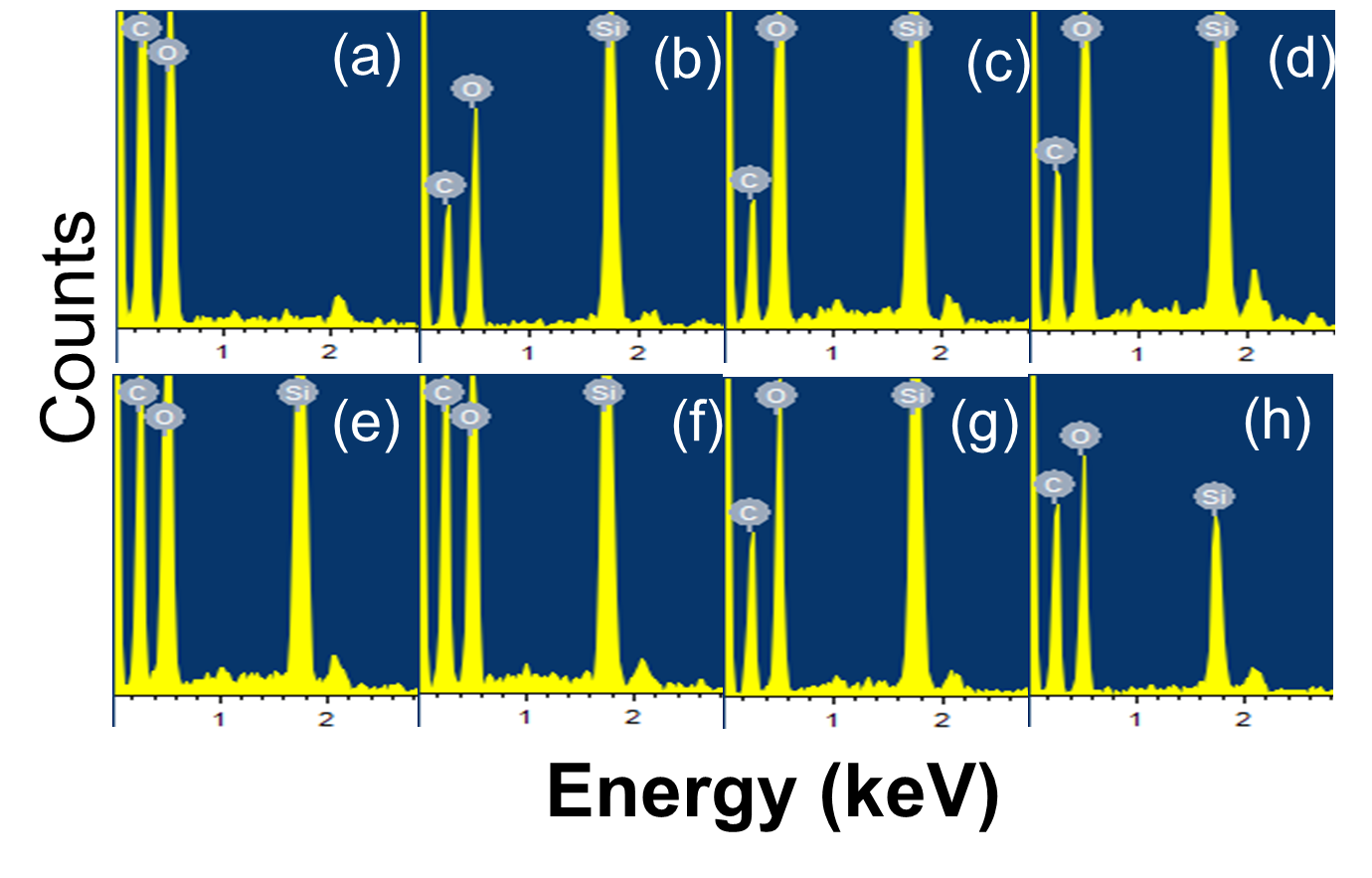


Figure S4. EDX spectra of unmodified and modified CF: CF_0:0_ (a), CF_1:0_ (b), CF_4:1_ (c), CF_3:2_ (d), CF_1:1_ (e), CF_2:3_ (f), CF_1:4_ (g), and CF_0:1_ (h).








Figure S5. Wide scan of XPS spectra of unmodified and modified CF (a) and high-resolution spectrum for Si 2p of CF_1:1_ (b).





Figure S6. TGA thermograms of the CF and CF_3:2_


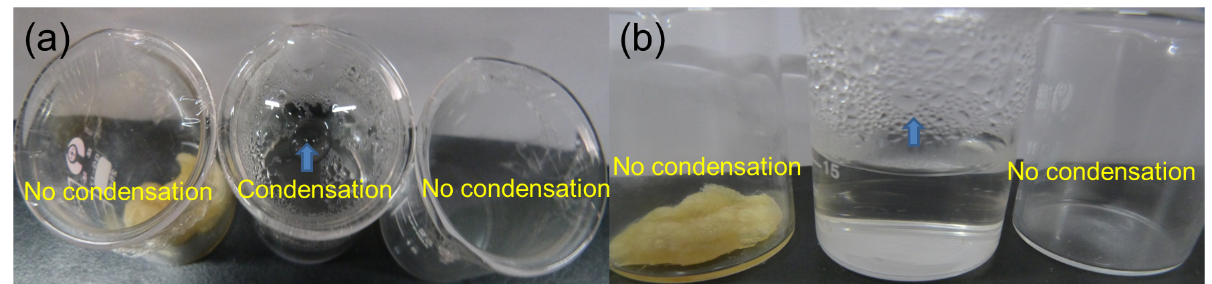


Figure S7. Selectivity test of oil absorption by CF_3:2_.


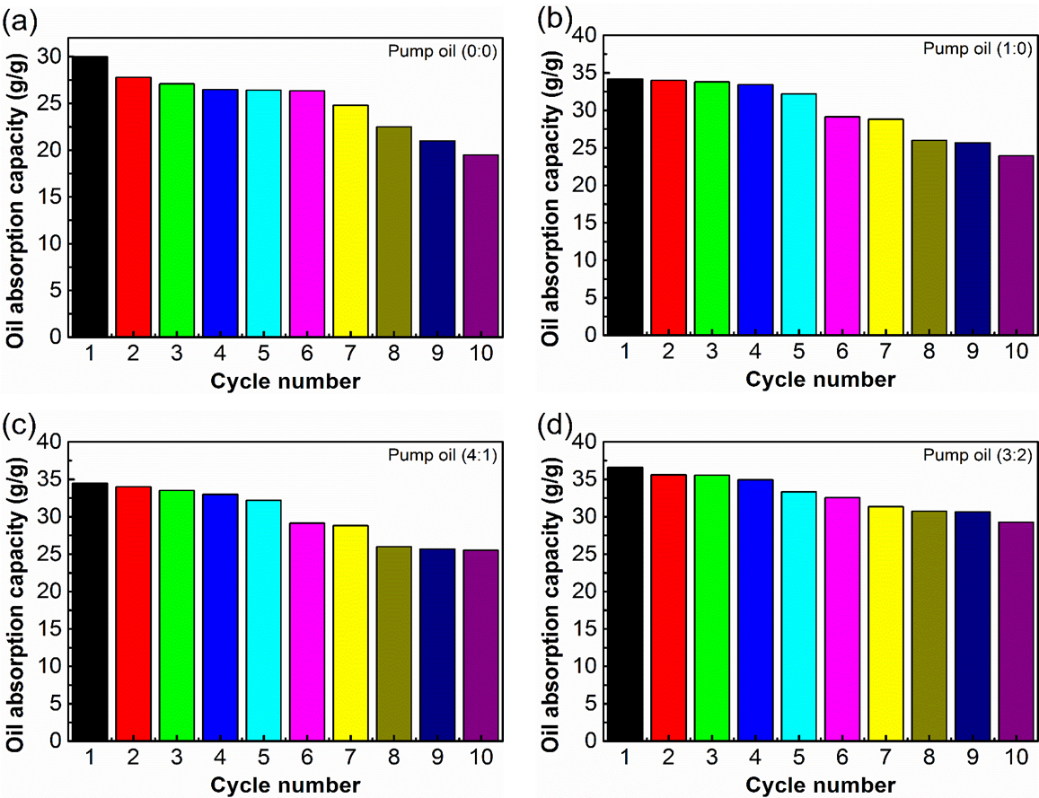


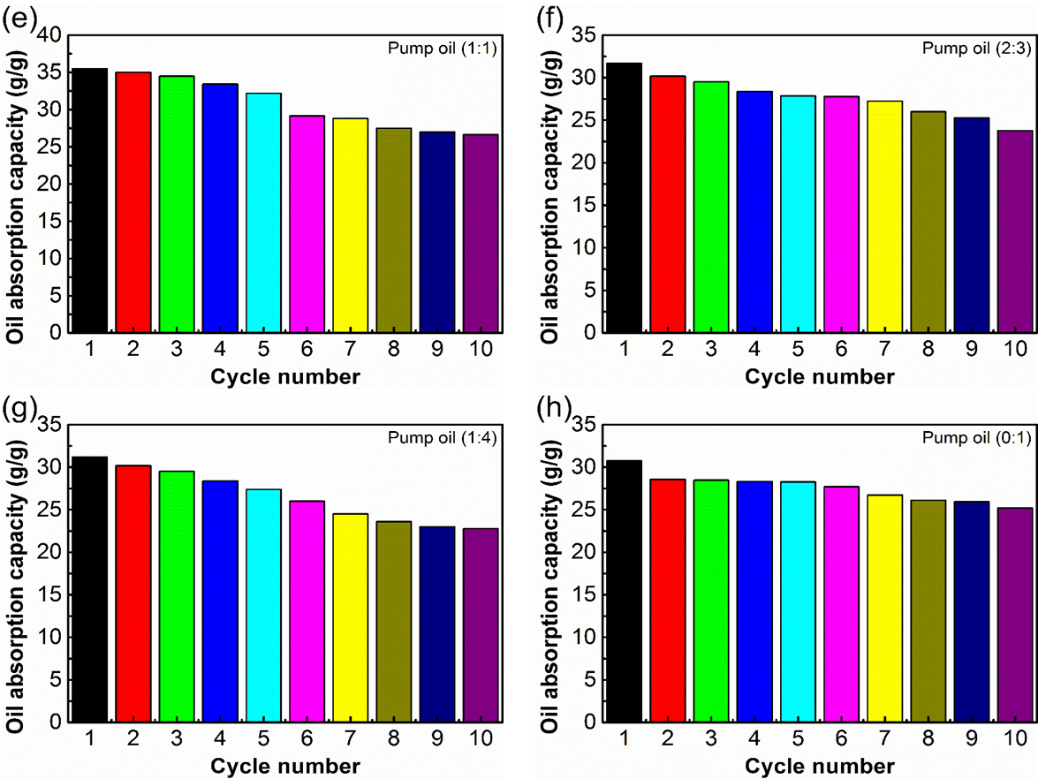


Figure S8. Pump oil absorption of unmodified and modified CF: CF_0:0_ (a), CF_1:0_ (b), CF_4:1_ (c), CF_3:2_ (d), CF_1:1_ (e), CF_2:3_ (f), CF_1:4_ (g), and CF_0:1_ (h).


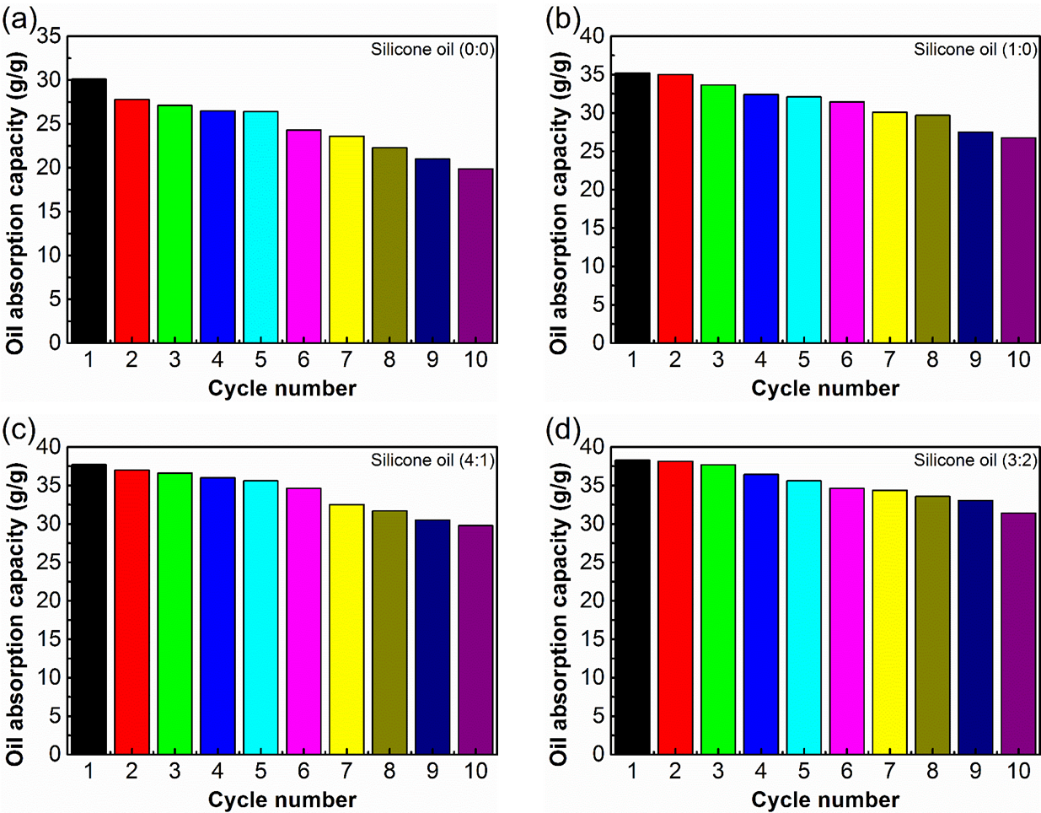


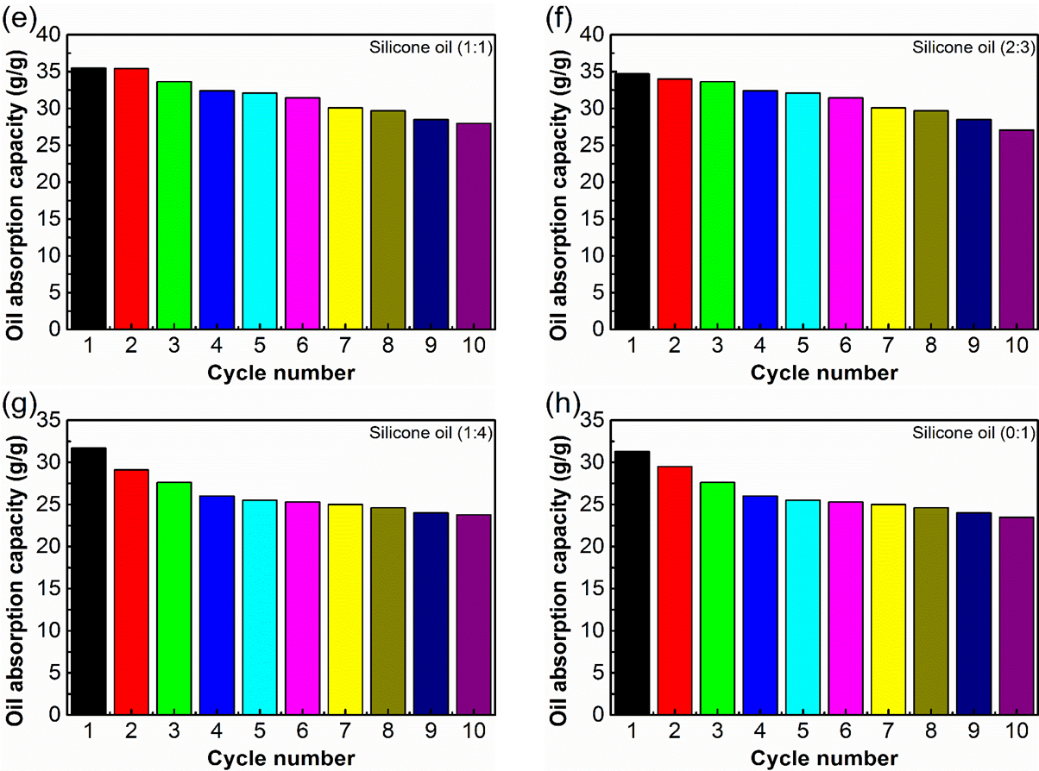


Figure S9. Silicone oil absorption of unmodified and modified CF: CF_0:0_ (a), CF_1:0_ (b), CF_4:1_ (c), CF_3:2_ (d), CF_1:1_ (e), CF_2:3_ (f), CF_1:4_ (g), and CF_0:1_ (h).

Table S1. Recycling of used cigarette filters


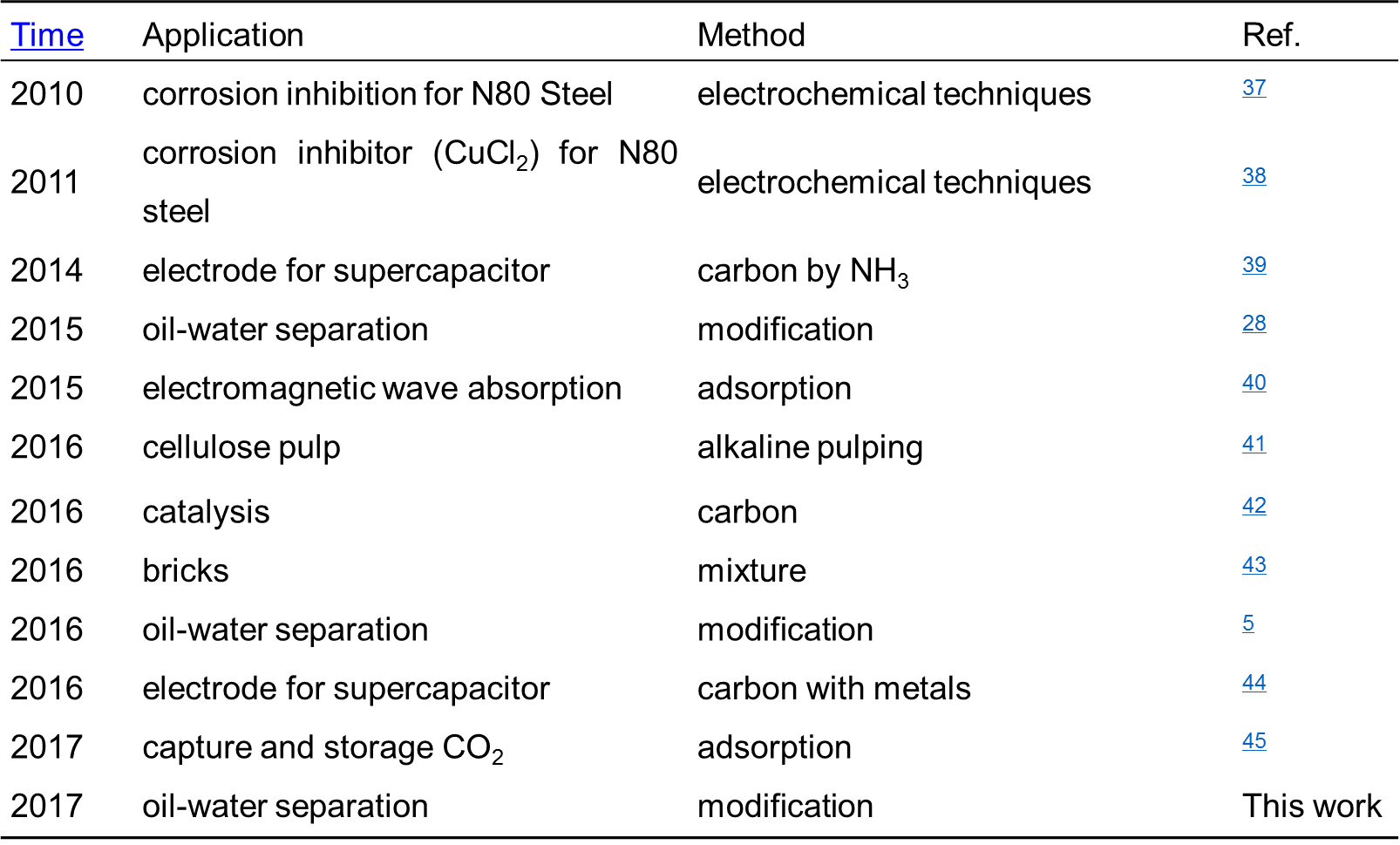

Supplement: Supplementary file 1 [file polymers-10-01101-s001.zip › Supporting Information 2018.09.19.docx]
